# Detection of novel insect flavivirus sequences integrated in *Aedes albopictus *(Diptera: Culicidae) in Northern Italy

**DOI:** 10.1186/1743-422X-6-93

**Published:** 2009-07-05

**Authors:** David Roiz, Ana Vázquez, Mari Paz Sánchez Seco, Antonio Tenorio, Annapaola Rizzoli

**Affiliations:** 1 IASMA Research and Innovation Centre, Environment and Natural Resources Area, Edmund Mach Foundation, S. Michele all'Adige (TN), Italy; 2 Laboratorio de arbovirus y enfermedades víricas importadas. Centro Nacional de Microbiologia, Instituto de Salud Carlos III, Madrid, Spain

## Abstract

The presence of DNA sequences integrated from a new flavivirus related to Cell Fusing Agent and Kamiti River Virus was identified in wild *Aedes albopictus *mosquito populations from the provinces of Trentino and Padova, Northern Italy. Field work was developed during August–October 2007 with BG-traps, and mosquitoes were screened for flavivirus and alphavirus. No alphavirus was detected, indicating that Chikungunya virus is not present in these mosquitoes in Trentino and Padova area. However, 21% of the pools were positive for flavivirus, further recognised with BLAST as similar to Kamiti River Virus. Phylogenetical analysis with 708 nucleotides from the NS5 gene identified this virus as a new member of the insect flavivirus clade, together with others like Kamiti River Virus, Cell Fusing Agent or *Culex *flavivirus, and in the group of those transmitted by *Aedes*. Furthermore, the treatment with RNAse, indicated that this flavivirus should be integrated in the genome of *Ae. albopictus*. These results propose that these sequences are transmitted by both sexes, and with different prevalence in the studied populations, and support the idea of a widespread distribution of integrated genomes in several mosquitoes from different areas, as first demonstrated with Cell Silent Agent. Evolutionary implications of this discovery and application in flavivirus phylogeny are discussed.

## Findings

The Asian tiger mosquito, *Aedes albopictus*, is a competent vector of more than 20 arboviruses, such as Dengue, and has been the principal vector of Chikungunya around the Indian Ocean, India and recently Italy [1]. Apart from Chikungunya, an outbreak of West Nile virus has been reported in Northern Italy [2,3]. Within the genus *Flavivirus*, some viruses belonging to the insect flavivirus group propagate only in mosquito cells and not in mammal cells. They may represent a basal lineage of the genus that diverged from other flaviviruses before the separation of the mosquito- and tick-borne groups [4]. Cell Fusing Agent virus (CFAV) isolated from *Aedes aegypti *and Kamiti River virus (KRV), isolated from *Aedes macintoshi *[5], were the first insect flaviviruses to be described. *Culex *flavivirus (CxFV) was isolated from *Culex pipiens *in Japan and Indonesia [6], and from *Culex quinquefasciatus *in Guatemala [7].

Recently, a DNA sequence named Cell Silent Agent (CSA), related to the NS1-NS4A genes of CFAV and KRV, was identified in an uninfected *Ae. albopictus *C6/36 cell line and in wild and laboratory-bred mosquitoes from different areas of the world [8]. This mechanism for the capture of genomic information from a non-retroviral RNA virus is also described in other flaviviruses, such as Tick-Borne Encephalitis virus [9]. Several flaviviruses, phleboviruses and DNA sequences of insect flaviviruses have been recently described in mosquitoes and sand flies from Spain [10].

Mosquitoes were collected by BG-traps (BioGents, Germany) at Arco and Riva del Garda, (Trento) and Padova, Italy, during August–October 2007. Each individual mosquito was identified and pooled according to species, sex, locality and date with a maximum of 50 adults per pool. A volume of 560 μl of AVL carrier/buffer RNA solution (Qiagen) was added to each pool before being conserved at -80°C. RNA was extracted with QIAamp Viral RNA Mini Kit (Qiagen). A combination of West Nile virus (strain Eg-101) and Ross River virus (strain T-48) was used as a positive control. A nested PCR was performed for all RNA extracts using degenerate primers targeting flavivirus [11] and alphavirus [12] in a Peltier Thermal cycler PCT-200 (MJ Research, Watertown, MA, USA). Positive PCR products were purified with a QIAquick PCR Purification Kit and a QIAquick Gel Extraction Kit (Qiagen), the latter for unspecific bands. Several samples were cloned with a Topo-TA Cloning Kit (Invitrogen). Amplified products (143 bp for flavivirus, and 195 bp for alphavirus) were further sequenced with ABI Prism BigDye Terminator Cycle sequencer v3.1 ready reaction (Applied Biosystems) and analyzed with the ABI PRISM 377 DNA Analyzer (Applied Biosystems). Sequence analysis was carried out with EditSeq and SeqMan software (DNASTAR Inc.). The relatedness of these sequences to the databases was assessed with the Basic Local Alignment Search Tool, implemented using the NCBI against the complete GenBank database.

**Figure 1:**
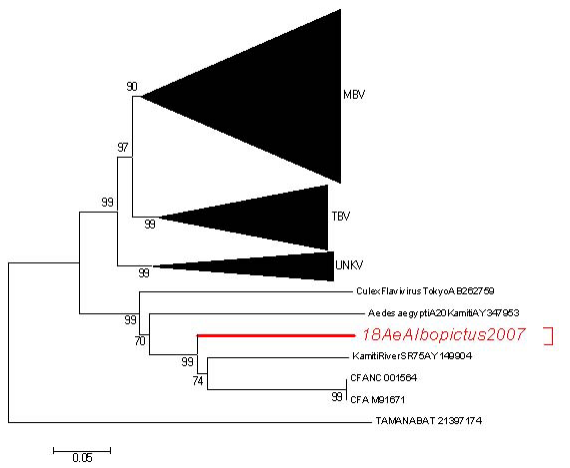
**Phylogenetic tree based on the NS5 protein region of 154 flaviviruses using the neighbor-joining method with Mega 3.1**. Bootstrap values correspond to 1000 replications. MBV: Mosquito-borne viruses, TBV: Tick-borne viruses, UNKV: Unknown vector, CFAV: Cell Fusing Agent, TAMANABAT: Tamana virus (as an outgroup). Flaviviruses used in the phylogenetic study (Accession Number): GenBank: AF202541, DQ118127, AY278441, AY278442, AF260968, D00246, AY274505, DQ256376, AY688948, DQ116961, DQ318020, AY277251, AY765264, AF013384, AF013413, AF013360, AY898809, AF013360, AF013389, AF161266, AY453411, NC006551, AF013412, AY453412, AF013367, AB241119, AF221499, M18370, NC001437, EF107523, AF486638, AB196925, AF013375, AY632538, AF013362, AF013390, AF013366, AY632536, DQ525916, AY632544, NC007580, DQ525916, AF013397, AY632542, AB110485, AF013376, AY632539, AB110489, AB026994, AF013392, AF013377, AF013363, AY632535, AF013406, AY632540, AF013382, AF119661, DQ181799, AY702040, U88535, DQ285561, AY708047, AF298807, AY762084, AY776330, AY762085, AY618988, AF100466, AY858044, AY858047, AY744685, AF013407, AF013383, AY632541, NC009029, DX03700, NC002031, U21055, AY968065, AY968064, AY632543, DQ837642, AF013372, AF013364, AF013411, VL40951, AF013378, AF013400, AF013395, DQ837641, AY632537, AF013373, AF013414, AF013405, AF013386, DQ235144, AF013403, DQ235150, AF013410, DQ235148, AF013380, DQ235146, AF013374, DQ235145, AF013398, DQ235149, AF310943, AF310941, AF311056, DQ462443, AF013381, AF013385, AY323490, AF331718, NC003690, AF253419, S35365, AY193805, AY438626, NC005062, DQ989336, AF013399, AY217093, DQ235151, DQ235153, NC001672, U39292, AF527415, DQ235152, AF013391, AY07863, AF013415, AF013402, AF160193, AF013401, AF013370, AF013387, AJ242984, AJ299445, AF013388, AF013396, AF144692, AF013371, AF013365, AF013368, AF013394, AF013369, AY347953, AB262759, NC_001564, M91671, AY149904, NC_003996.

A RT-PCR for the NS5 gene that codifies for the non-structural 5 protein (A. Vazquez, unpub. data) was used to clarify the phylogenetic relationships between the detected flaviviruses. The phylogenetic analysis covered a region of 708 pb and 154 flavivirus sequences obtained from GenBank (Fig. 1). The neighbour-joining method and distance-p model were used to construct a tree with the MEGA 3.1 software [13] with 1000 replications for obtaining the bootstrap values. The phylogenetic tree (Fig. 1) was reconstructed using alignments of the amino acid sequences and the nucleotide sequences.

In order to verify that positive pools were the result of RNA amplification and not of integrated DNA, 5 μl from different positive samples was digested with 2 μl of bovine pancreas RNAse (Sigma) and incubated for 2 h at 37°C [10]. We applied a generic multiplex RT-nested-PCR for flavivirus and phlebovirus with internal control of the phlebovirus Toscana, derived from previously published methods [10-12,14], and these treated extracts were directly amplified without a previous retro-transcription step. In a parallel analysis, each of these positive aliquots was assayed by RT-nested-PCR for flavivirus using 5 μl of untreated extracts to confirm that RNA was not degraded.

Out of the 969 mosquitoes collected, 727 (75%) were females, distributed over a total of 32 pools of 3 to 50 individuals each (Table 1). None of the pools was positive for alphavirus or West Nile, indicating that Chikungunya and the West Nile virus were not present in these mosquitoes. However, 21% (a total of 7 pools) were positive for flavivirus amplification and showed similarities with KRV using BLAST software. Phylogenetic reconstruction of the seven NS5 sequences using 154 homologous sequences of flavivirus, with robustness bootstrap values, identified this insect flavivirus as a new group; separate from KRV and CFAV but in the same clade (Fig. 1). These results bore strong similarities with other classifications [4] and confirmed the evolutionary relationship of this insect virus with KRV and CFAV. The percentages of identity between the sequences found in this study and KRV, CFAV and CxFV were 73.3%, 72% and 62,1% respectively in nucleotides and 86.9%, 84.3% and 63.6% respectively at the amino acidic level. Therefore, this virus clearly belongs to the insect flavivirus clade, but is more closely related to KRV and CFAV than to *Culex *flavivirus. As these sequences could have been either RNA or DNA [8,10], further treatment with RNAse allowed for subsequent PCR amplification. The results showed that these sequences are DNA molecules and not RNA and provide evidence for integration into the mosquito genome.

**Table 1:** List of analysed pools and positive results for the flaviviruses identified as a novel insect flavivirus.

Sample ID	Flavivirus	Locality	Province	Mosquito species	Sex	Number of mosquitoes
1	+	Riva 1	TN	*Aedes albopictus*	F	3

2	-	Riva 1	TN	*Culex pipiens*	F	45

2B	-	Riva 1	TN	*Culex pipiens*	M	25

3	-	Riva 2	TN	*Aedes albopictus*	F	50

3B	+	Riva 2	TN	*Aedes albopictus*	F	50

5	-	Arco 1	TN	*Aedes albopictus*	F	31

6	+	Arco 1	TN	*Aedes albopictus*	M	37

12	-	Arco 2	TN	*Aedes albopictus*	F	40

13	+	Arco 2	TN	*Aedes albopictus*	M	25

14	-	Arco 3	TN	*Aedes albopictus*	F	35

14B	-	Arco 3	TN	*Aedes albopictus*	F	32

15	-	Arco 3	TN	*Aedes albopictus*	M	15

17	-	Riva 3	TN	*Aedes albopictus*	F	12

18	+	Riva 3	TN	*Aedes albopictus*	M	7

20	-	Arco 2	TN	*Aedes albopictus*	F	6

21	-	Arco 2	TN	*Aedes albopictus*	F	37

22	-	Arco 2	TN	*Aedes albopictus*	F	48

23	-	Arco 2	TN	*Aedes albopictus*	F	49

24	-	Arco 2	TN	*Aedes albopictus*	M	3

26	-	Arco 3	TN	*Aedes albopictus*	F	42

27	-	Arco 3	TN	*Aedes albopictus*	F	50

28	-	Arco 3	TN	*Aedes albopictus*	F	50

29	-	Arco 3	TN	*Aedes albopictus*	F	46

30	-	Arco 3	TN	*Aedes albopictus*	F	50

31	-	Arco 3	TN	*Aedes albopictus*	M	25

32	-	Arco 3	TN	*Aedes albopictus*	F	28

32B	-	Arco 3	TN	*Aedes albopictus*	M	12

33	-	Padova	PD	*Aedes albopictus*	F	45

34	+	Padova	PD	*Aedes albopictus*	M	17

35	-	Padova	PD	*Aedes albopictus*	F	21

36	+	Padova	PD	*Aedes albopictus*	M	4

37	-	Arco 4	TN	*Aedes albopictus*	F	39

TN: province of Trento, PD: province of Padua.

It has been suggested that the genus *Flavivirus *may include a large number of species yet to be identified and several of them could be insect flaviviruses, as proved by this work and other recent field investigations in different areas of the world [5-8,10,15]. Based on the data reported in this study, we consider these sequences to be a new group belonging to the insect flavivirus clade and integrated into the *Ae. albopictus *genome. The cluster of these sequences, which are more closely related to insect flaviviruses associated with *Aedes *(CFAV, KRV) than to the *Culex *flavivirus (CxFV), suggests that there is host-virus specificity within the insect flavivirus clade and that these groups have evolved independently [6]. This result supports the existence of several flavivirus-related sequences integrated into the DNA of several mosquito species in several locations in the world, as well as the existence of several integration events [6,8]. Integration events have been described only in *Aedes *species, but could also be described in other genera, such as *Culex*, and other arthropods, such as ticks. These sequences could be integrated into the genes of *Ae. albopictus *following infection by the corresponding flavivirus (that has yet to be discovered), and may be a source of evolution for *Ae. albopictus *mosquitoes, representing a different mechanism with which genetic diversity may be generated in eukaryotic cells [8].

As mosquito-borne viruses are supposed to have evolved from insect flavivirus, further analysis of these results and of the other insect flaviviruses will help to clarify the nature, origin and evolution of the flavivirus genus. It is important to analyze more thoroughly whether the presence of insect flavivirus infecting *Ae. albopictus *and other arthropod vectors could interfere with infection by other arboviruses, subsequently altering the transmission capacity of certain vector populations for several vector-borne diseases.

## Competing interests

The authors declare that they have no competing interests.

## Authors' contributions

DR designed the study, carried out the field work, the detection of flavivirus, the analysis and drafted the paper. AV made the phylogenetic analysis and the RNAse probes and drafted the paper, MPSS participated in the sequence alignment and drafted the paper, AT participated in the design of the study and helped to draft the paper, AR coordinated the work and helped to draft the paper. All authors read and approved the final manuscript.
